# A Case of Mucosal Cancer of the Stomach Treated by Endoscopic Submucosal Dissection after Which Nodal Metastasis Became Evident

**DOI:** 10.1155/2013/853849

**Published:** 2013-02-27

**Authors:** Takashi Obana, Naotaka Fujita, Yutaka Noda, Dai Hirasawa, Kei Ito, Toshiki Sugawara, Yoshihiro Harada, Tetsuya Oohira, Hiroshi Honda, Takashi Sawai

**Affiliations:** ^1^Department of Gastroenterology, Sendai City Medical Center, 5-22-1, Tsurugaya, Miyagino-ku, Sendai 983-0824, Japan; ^2^Department of Surgery, Sendai City Medical Center, Sendai 983-0824, Japan; ^3^First Department of Pathology, Iwate Medical University, Morioka 983-0824, Japan

## Abstract

An 82-year-old male was referred to our institution for evaluation and treatment of a protruding lesion in the stomach. Esophagogastroduodenoscopy (EGD) showed a small protruding lesion and a large superficial elevated lesion on the lesser curvature of the stomach (macroscopic type: 0-I and 0-IIa, resp.). CT and endoscopic ultrasonography (EUS) visualized a small round lymph node (LN) 11 mm in size near the lesser curvature, although submucosal invasion was not evident. These two lesions were resected en bloc by endoscopic submucosal dissection (ESD). Pathological examination of the resected specimen showed moderately differentiated tubular adenocarcinoma (tub2) and well-differentiated tubular adenocarcinoma (tub1), respectively, which were limited to the mucosal layer. Because lymphatic-vascular involvement was not detected by hematoxylin and eosin (HE) staining, additional gastrectomy was not performed. Two months after ESD, follow-up EUS and CT showed an enlarged LN. EUS-guided fine needle aspiration (EUS-FNA) for the LN revealed metastasis. Therefore, total gastrectomy with LN dissection was performed. His postoperative course was uneventful. After discharge, he has been followed up at the outpatient department without any sign of recurrence for 5 years. Histological reexamination of the ESD specimen using immunohistochemistry showed lymphatic invasion of cancer cells in the lamina propria of the 0-I lesion 13 mm in size.

## 1. Introduction

 With the introduction of endoscopic submucosal dissection (ESD), curative resection has become achievable even for large early gastric cancers (EGCs) if the depth of invasion is limited to the mucosal layer of the gastric wall. However, approximately 2% of mucosal cancer of the stomach can metastasize to lymph nodes (LNs) [1]. Ulcer formation, histological findings of undifferentiated carcinoma, tumor size above 3 cm, lymphatic-vascular involvement, and so forth are recognized as risk factors for LN metastasis in intramucosal EGCs [1, 2]. Vigorous histological examination and postoperative periodical followup are necessary to avoid cancer-related death. Herein, we present a case of small intramucosal EGC in which LN metastasis was proven after ESD by endosonography-guided fine-needle aspiration (EUS-FNA). 

## 2. Case Report

**Figure 1:**
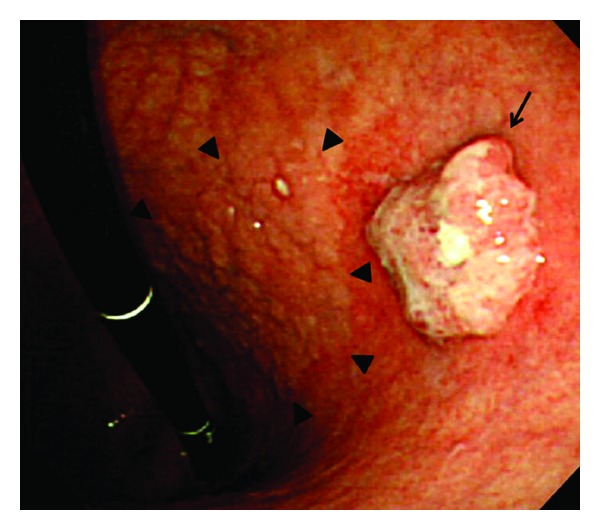
EGD showed a small protruded lesion (0-I) on the lesser curvature of the lower gastric body (arrow), and a large superficial elevated lesion (0-IIa) on the lesser curvature of the stomach which spread from the upper to the lower gastric body was also visualized (arrowheads).

 An 82-year-old male with concomitant chronic obstructive pulmonary disease (COPD) was referred to our institution for further evaluation and treatment of a protruded lesion in the stomach. His physical examination on admission was unremarkable. Laboratory data, including CEA and CA19-9, were almost normal. EGD showed a small protruding lesion on the lesser curvature of the lower gastric body, and also a large superficial elevated lesion on the lesser curvature of the stomach which spread from the upper to the lower gastric body (Figure 1). Biopsy specimens taken from the lesions showed moderately differentiated tubular adenocarcinoma (tub2) and well-differentiated tubular adenocarcinoma (tub1), respectively. EUS and CT visualized a small round LN 11 mm in size located near the lesser curvature side of the stomach, although submucosal invasion was not evident. The patient chose to undergo ESD first under detailed informed consent.

ESD was performed with a hook knife, and the two lesions were resected en bloc. The resected specimen included a small protruded lesion (0-I) 13 × 10 mm in size and a large superficial elevated lesion (0-IIa) 89 × 33 mm in size.

**Figure 2:**
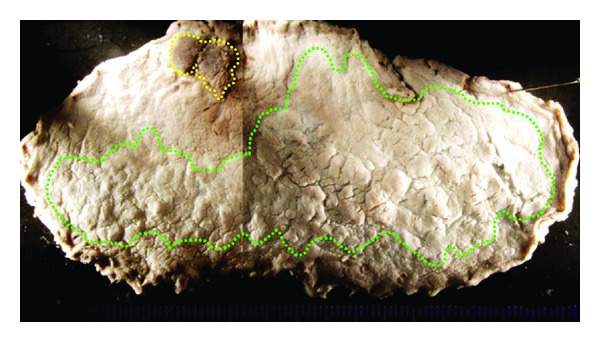
Formalin-fixed endoscopically resected specimen. The yellow-dotted line and the green-dotted line indicate the extent of the 0-I lesion and the 0-IIa lesion, respectively. The intervening mucosa between the two lesions was nonneoplastic.

Histological examination of the two lesions showed tub2 and tub1, respectively. The intervening mucosa between the two lesions was nonneoplastic (Figure 2). The depth of tumor invasion was limited to the mucosal layer, and lymphatic-vascular involvement was not evident by HE staining in either lesions. Based on these findings, additional gastrectomy was not performed. 

He underwent follow-up EGD/EUS and CT 2 months after ESD. No residual cancerous lesion was observed in the stomach. On the other hand, the LN became enlarged from 11 mm to 16 mm in size as measured by EUS. EUS-FNA with the cell block method revealed LN metastasis of poorly differentiated adenocarcinoma (por). Subsequently, total gastrectomy with LN dissection was performed 4 months after ESD. Histological examination of the surgically resected specimen revealed metastasis to the No. 3 LN. No residual cancer was detected in the stomach. His postoperative course was uneventful. After discharge, he has been followed up at the outpatient department without any sign of recurrence for 5 years. 

**Figure 3:**
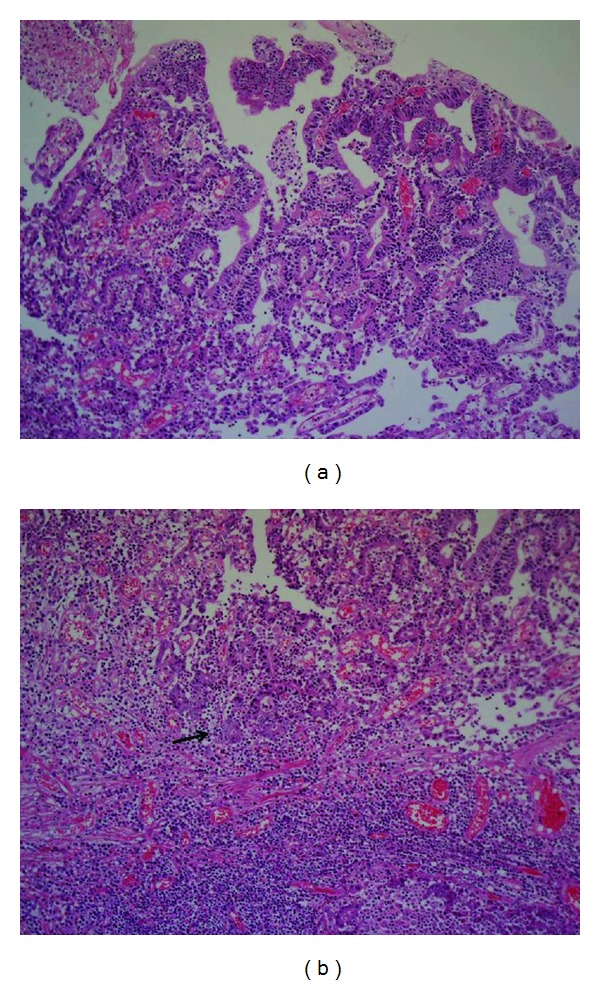
(a) Surface of the 0-I lesion showed tub2 (HE ×25). (b) Tumor was poorly-differentiated at the front of intramucosal invasion (arrow: HE ×25).

**Figure 4:**
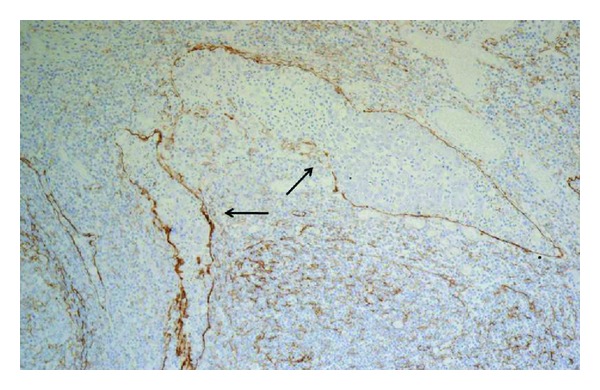
Lymphatic invasion is evident by D2-40 staining in the lamina propria (arrows: ×25).

**Figure 5:**
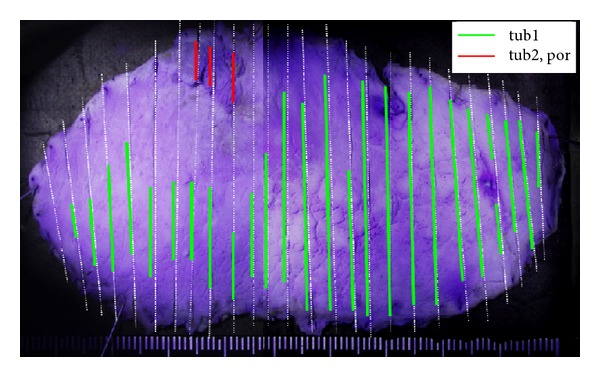
Final histological mapping of the endoscopically resected specimen after crystal violet staining.

Reexamination of the specimen obtained by ESD was carried out with special staining and immunohistochemistry. Smooth muscle actin (SMA) staining revealed that submucosal invasion was not present in either lesions. However, tumor cells became poorly differentiated at the front of the mucosal invasion in the 0-I lesion (Figures 3(a) and 3(b)), and D2-40 staining revealed lymphatic invasion in the lamina propria (Figure 4). Vascular involvement was not detected by either factor-VIII staining or Elastica-Masson (EM) staining. The mucin expression pattern of the 0-I lesion was MUC1(−), MUC2(−), MUC 5AC(+), and MUC6(−), suggesting a gastric-type mucin phenotype. On the other hand, lymphatic-vascular involvement was not proven by D2-40, factor-VIII, or EM staining in the large 0-IIa lesion. Finally, the lesions were pathologically diagnosed as 0-I, tub2 > por, 13 × 10 mm, pT1(M), ly2, v0, INF *β*, VM(−), LM(−); and 0-IIa, tub1, 89 × 33 mm, pT1(M), ly0, v0, VM(−), LM(−), respectively (Figure 5). 

## 3. Discussion 

 Since the introduction of ESD, the number of endoscopic resections for early EGCs has been increasing. The accepted extended indications for ESD are as follows: (1) differentiated mucosal cancer without ulcer and of any size; (2) differentiated mucosal cancer, with ulcer, and ≤3 cm in size; and (3) differentiated submucosal cancer (sm1, i.e., <500 *μ*m penetration into the submucosa) without lymphovascular invasion and ≤3 cm in size [1, 3]. Recently, it has been shown that undifferentiated-type mucosal EGCs 20 mm or less in size without lymphatic-vascular involvement and ulcerative findings present a negligible risk of LN metastasis (95% confidence interval, 0–0.96%) and could be a possible indication for ESD [4].

However, several problems remain to be resolved. For example, the rate of LN metastasis of differentiated/undifferentiated mixed-type EGC has not been fully investigated in large case series. The other problem is that the diagnosis of lymphatic invasion is sometimes difficult with conventional HE staining. A selective marker of lymphatic epithelium, D2-40, is a useful tool for detecting lymphatic vessels. In a previous study [5], D2-40 positive lymphatic vessels were observed in the deeper lamina propria layer, and the lymphatic vessel density was high in the muscularis mucosa layer. These lymphatic vessels located in the mucosal layer can be a pathway for LN metastasis. Although D2-40 staining is proven to increase the accuracy of lymphatic involvement [5], it is not practical to perform D2-40 staining for all resected specimens. Therefore, periodical followup after ESD is mandatory for early detection of LN metastasis, especially when the lesion was finally diagnosed as mixed-type EGC even if curative resection was confirmed with HE staining. 

The present case is considered to be relatively rare in which LN metastasis developed in a small mucosal cancer (0-I). Initially, EUS and CT visualized a small round LN 11 mm in size before ESD. When the criteria for diagnosing LN metastasis are LNs which are 1 cm or larger in size, and hypoechoic or round instead of elliptical by EUS, the pooled sensitivity and specificity of EUS for diagnosing LN metastasis are reported to be 58.2% (95% CI: 53.5–62.8) and 87.2% (95% CI: 84.4–89.7), respectively [6]. Another systematic review article [7] has reported that the median sensitivity and specificity of assessing LN metastasis by EUS, MDCT, MRI, and FDG-PET are 70.8% and 84.6%, 80.0% and 77.8%, 68.8% and 75.0%, and 34.3% and 93.2%, respectively. Although the possibility of LN metastasis was not ruled out, total gastrectomy was considered to pose some risk for an 82-year-old patient with COPD. Therefore, we consider that our strategy of performing ESD first was acceptable from a practical point of view. The 0-I lesion was histologically diagnosed as mucosal cancer (the lamina propria) without ulcerative change. The rate of LN metastasis of mucosal cancer without an ulcer is reported to be 1.4%. However, for cases with lymphatic-vascular involvement, the rate rises to 26.3% [1]. 

Considering the fact that the swollen LN was detected by preoperative imaging studies, additional histological examination after special staining and immunohistochemistry for the endoscopically resected specimen and/or EUS-FNA prior to ESD should have been performed 

One of the characteristic features of the lesion was that the tumor became poorly differentiated (por) at the front of intramucosal invasion (i.e., sprouting). The present case suggests that if sprouting is observed by HE staining, immunohistochemistry is recommended to rule out lymphatic-vascular invasion even, if the depth of invasion is limited to the mucosal layer. 

Another interesting histological feature was the mucin phenotype. Several articles [8, 9] have suggested that differentiated EGCs with gastric-type mucin phenotype tend to show mixed-type histology (differentiated-type carcinoma with an undifferentiated-type carcinoma component) and LN metastasis. These findings were concordant with our case. 

In the present case, EUS-FNA was useful for obtaining histological evidence of LN metastasis preoperatively. The cell block method with immunostaining has been shown to improve diagnostic yield more than smear cytology in patients who undergo EUS-FNA without on-site cytology [10]. However, the safety and effectiveness of EUS-FNA for perigastric LNs of gastric cancer patients should be established by a substantial number of studies because there might be some risk of tumor cell dissemination. 

 In summary, we experienced a case of small mucosal cancer in which LN metastasis became evident after ESD. Lymphatic invasion in the lamina propria was confirmed by D2-40 staining. Close postoperative followup and EUS-FNA for the swollen perigastric LN were useful in establishing diagnosis of LN metastasis.
